# Tactile Avatar: Tactile Sensing System Mimicking Human Tactile Cognition

**DOI:** 10.1002/advs.202002362

**Published:** 2021-02-08

**Authors:** Kyungsoo Kim, Minkyung Sim, Sung‐Ho Lim, Dongsu Kim, Doyoung Lee, Kwonsik Shin, Cheil Moon, Ji‐Woong Choi, Jae Eun Jang

**Affiliations:** ^1^ Department of Information and Communication Engineering Daegu Gyeongbuk Institute of Science & Technology (DGIST) Daegu 711‐873 Korea; ^2^ Department of Neurology University of California San Francisco (UCSF) San Francisco CA 94158 USA; ^3^ Department of Brain and Cognitive Sciences Daegu Gyeongbuk Institute of Science & Technology (DGIST) Daegu 711–873 Korea

**Keywords:** machine learning, P(VDF‐TrFE), piezoelectric effect, tactile avatars

## Abstract

As a surrogate for human tactile cognition, an artificial tactile perception and cognition system are proposed to produce smooth/soft and rough tactile sensations by its user's tactile feeling; and named this system as “tactile avatar”. A piezoelectric tactile sensor is developed to record dynamically various physical information such as pressure, temperature, hardness, sliding velocity, and surface topography. For artificial tactile cognition, the tactile feeling of humans to various tactile materials ranging from smooth/soft to rough are assessed and found variation among participants. Because tactile responses vary among humans, a deep learning structure is designed to allow personalization through training based on individualized histograms of human tactile cognition and recording physical tactile information. The decision error in each avatar system is less than 2% when 42 materials are used to measure the tactile data with 100 trials for each material under 1.2N of contact force with 4cm s^−1^ of sliding velocity. As a tactile avatar, the machine categorizes newly experienced materials based on the tactile knowledge obtained from training data. The tactile sensation showed a high correlation with the specific user's tendency. This approach can be applied to electronic devices with tactile emotional exchange capabilities, as well as advanced digital experiences.

## Graphical abstract

This article suggests a tactile avatar system as a surrogate for human tactile cognition producing smooth/soft and rough tactile sensation. Because tactile responses vary among humans, a deep learning network is designed considering the cognitive processing of independent tactile features engaged by humans. As a result, the tactile tendency of a specific user is successfully mimicked by this system.

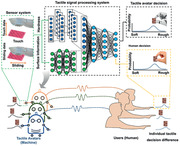

## 1 Introduction

Digital experiences based on the five human senses have improved with advancements in electrical devices and signal processing.^[^ ^1^ ^]^ For example, virtual reality (VR) provides visual and auditory sensations, and more personalized services can be supplied by augmented reality (AR), which delivers 3D spatial images and stereo sound.^[^ ^2^, ^3^ ^]^ Digital experiences are used in many fields, including entertainment and internet marketing.^[^ ^4^, ^5^ ^]^ Furthermore, these technologies are evolved in an attempt to exchange emotions between humans and machines. For a more immersive digital experience, sensing and delivering tactile information is necessary, where touching an object by hands is distinct from seeing and hearing. Therefore, considerable attention has been paid to technologies that provide tactile information, and various tactile sensors and actuators have been proposed and developed.^[^ ^6^, ^7^, ^8^, ^9^, ^10^, ^11^, ^12^, ^13^, ^14^, ^15^, ^16^, ^17^, ^18^ ^]^ However, unlike for vision and hearing, an artificial tactile or haptic system is limited in its ability to directly transfer physical values to humans or machines because of difficulties in generating tactile feelings.

Tactile sensation plays important role in our interactions with the external world, from both physical and emotional perspectives.^[^ ^19^ ^]^ On touching or sliding the fingers across an object, individuals may describe the tactile emotional sensation as “soft,” “hard,” “smooth,” “rough,” or even “painful”.^[^ ^20^ ^]^ Tactile receptors in human skin measure a unique feature of a tactile object, such as hardness, pressure, temperature, friction, or vibration.^[^ ^21^, ^22^ ^]^ Despite much information obtained by various receptors, the principal components of the tactile sensations experienced by humans can be described mainly as hardness, surface topography, and temperature.^[^ ^23^ ^]^ Therefore, an artificial sensor system detecting these parameters is required and the multimodal sensor system must obtain the same amount of physical information as a human does.^[^ ^24^, ^25^, ^26^, ^27^ ^]^ It is also important to consider the characteristics of human tactile perception as a standard for the development of an artificial tactile system.^[^ ^28^, ^29^ ^]^ So, several studies have conducted tactile experiments in humans to reveal human tactile perception.^[^ ^30^, ^31^, ^32^ ^]^ The material classification by tactile cognition has been studied to resemble the human brain processing.^[^ ^33^, ^34^, ^35^, ^36^ ^]^ The material classification ability of these systems, based on texture detection, has become similar to those of humans.^[^ ^37^ ^]^ Recently, deep learning has been employed in this field, especially to differentiate various types of materials, and it has improved the accuracy of material classification.^[^ ^38^, ^39^, ^40^ ^]^ Even, it was reported that the high classification performance of deep learning between two materials could be better than that of human tactile sensation.^[^ ^41^ ^]^ For using 117 textures, they maintained the performance of over 95.4%. In our previous study, we also conducted the research about the classification accuracy according to the number of active sensing cells.^[^ ^42^ ^]^ These researches related to sensing and classification of surface materials were useful to develop various haptic applications delivering the physical information to humans because the surface characteristics should be well recognized in advance.^[^ ^43^, ^44^, ^45^ ^]^ However, these tactile material classifications do not represent the overall tactile sensation of human. Because the processing of tactile feeling by the human brain has not yet been revealed clearly and tactile responses vary among humans, it is challenging to imitate the tactile sensation by an artificial system. Furthermore, because there are too many texture materials over the world, the limit of the previous classification method is clear. Therefore, like human tactile sensation, it is necessary to develop the tactile system deciding untrained surface materials based on trained data and generating a tactile feeling.

Herein, we report an artificial tactile system that can generate “smooth/soft” and “rough” tactile sensations in accordance with the user's tactile feeling, which we call a “tactile avatar”. Our tactile system has mimicked the psychological tactile feeling of the human based on a piezoelectric sensor system and deep learning process. First, we developed multiarray tactile sensors, with the same tactile resolution as that of the tactile receptors on human skin, to measure the principal physical components, that is, hardness, surface topography, and temperature^[^ ^46^ ^]^ required for the deep learning process. A piezoelectric sensor was used because of its advantages of self‐power generation, dynamic sensing, and temperature sensing abilities,^[^ ^47^, ^48^, ^49^ ^]^ similar to those of human skin receptors.^[^ ^50^ ^]^ Simple and intuitive analysis of the measured signal was performed before devising a deep learning network to investigate the surface information. The deep learning network was designed considering the cognitive processing of independent tactile features engaged by humans. Specifically, unlike other researches, we noted individual variation in scores assigned to materials in a tactile cognition experiment, in which the network was trained using the tactile histograms of individual participants. The human‐like sensor and processing system contributed to the artificial tactile cognition system of the tactile avatar. We analyzed the performance of the tactile avatar in terms of its tactile decision‐making. Additionally, we assessed its categorization performance for untrained, that is, novel, tactile materials.

## 2 Results and Discussion

### 2.1 Tactile Avatar Designed to Exhibit Human‐Like Tactile Classification Performance

**Figure 1:**
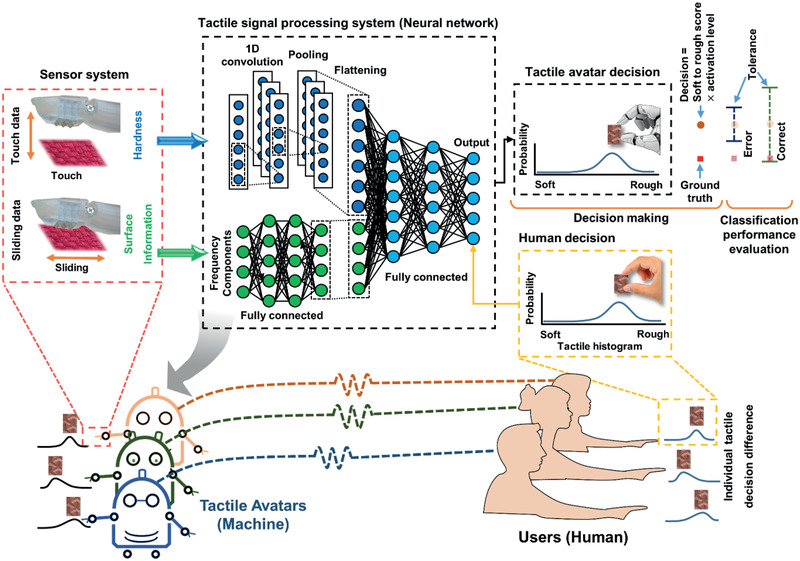
Tactile avatar system trained using the tactile decisions made by its human counterpart, capable of making tactile decisions similar to a human.

The human tactile system is complex,^[^ ^23^ ^]^ and the tactile information processing engaged in by humans has not yet been clarified in detail. Although it is not possible to fully model the sensing mechanism of the human tactile system, or process tactile feelings as accurate as a human using artificial means, fortunately, some principal physical components (i.e., hardness, surface topography, friction, and temperature) can adequately represent the fundamental mechanism of the human tactile system, and deep learning can be used to model the human neural network processes.^[^ ^39^, ^40^ ^]^ Some approaches have optimized the discrimination of tactile materials using artificial sensors; however, this does not mean that they are optimal for mimicking human cognition, because it pertains to distinguishing among tactile sensations. To represent this human tactile cognition, we designed a tactile avatar that includes a multiarray tactile sensor and a human cognition‐based deep learning network (**Figure** **1**). Moreover, for personalization, the tactile system was trained using individual tactile decision histograms. Figure 1 illustrates the mimicking by the avatar, of the tactile decisions made by its human counterpart, even for novel tactile materials.

To mimic the human tactile system, the cognitive processing part of the tactile avatar was based on deep learning networks. The first step in the process was to obtain the baseline signal arising from the touching and sliding processes in the parallel input layer in Figure 1. The multiarray sensors were made of piezoelectric materials having a high spatial resolution. The tactile sensor‐generated signals on the hardness, temperature, and surface topography of the tactile materials during sequential touching and sliding motions, similar to those made by humans on the surface of a tactile material. Although friction is an important parameter for tactile sensation because it shows a high correlation with surface topography,^[^ ^51^ ^]^ we do not consider it as a major parameter in the deep learning process. The details of the fabrication and measurement process by the tactile sensor were demonstrated in the Experimental section and Figure S1, Supporting Information.

The slope of the touch signal and oscillation frequency of the sliding signal contained information regarding the hardness and surface topography of the materials, respectively. To discriminate the tactile materials based on the signal arising from the developed sensors, a decision processing system was built for the tactile avatar using a combination of neural network layers, to emphasize certain features and allow for sample classification. The two different types of input data, that is, touching and sliding data, were processed separately in the hidden layer. To measure the slope of the touch signal, a 1D convolutional layer with a kernel size of four was used to capture the signal, which was then down‐sampled by the max pooling layer (because the peak touch signal in the time domain provided hardness information for the tactile sample). In parallel, the sliding signal was converted to the frequency domain by Fourier transform, because the surface topography was represented by frequency components.^[^ ^41^, ^52^, ^53^ ^]^ Two fully connected layers after Fourier transform were designed to allow more classification computations pertaining to the sliding signal to be added to the network, because the differences of the sliding signal in tactile samples are based on a complex combination of frequency components. The two processed tactile signals were combined and exposed to the two fully connected layers for complex discrimination in the hyper dimension, before being terminated with a SoftMax kernel. Note that the samples in the output layer were sorted from ‘smooth/soft’ to ‘rough’. Unlike typical supervised learning networks that label candidates from a predefined list, the last layer of our network used a histogram of tactile decisions made by a human participant. The last layer of the network used the histogram to assign multiple labels with different weights that reflected human tactile cognition. This improves the tactile learning capabilities of the machine because the potential combinations of weighted labels are unlimited. The personalized tactile avatar was characterized based on the scores assigned to tactile materials, which were ranked from the smoothest/softest to the roughest. The tactile avatar was trained using the tactile decision data of a human participant, so that it could make tactile decisions similar to those of that participant. The avatar could find applications in various fields such as online shopping, and VR and AR environments, that is, whenever tactile sensations may be desirable. Additionally, if trained using the data of several individuals, an artificial skin system can resemble the general human tactile feeling.

### 2.2 Tactile Decision‐Making by Humans

**Figure 2:**
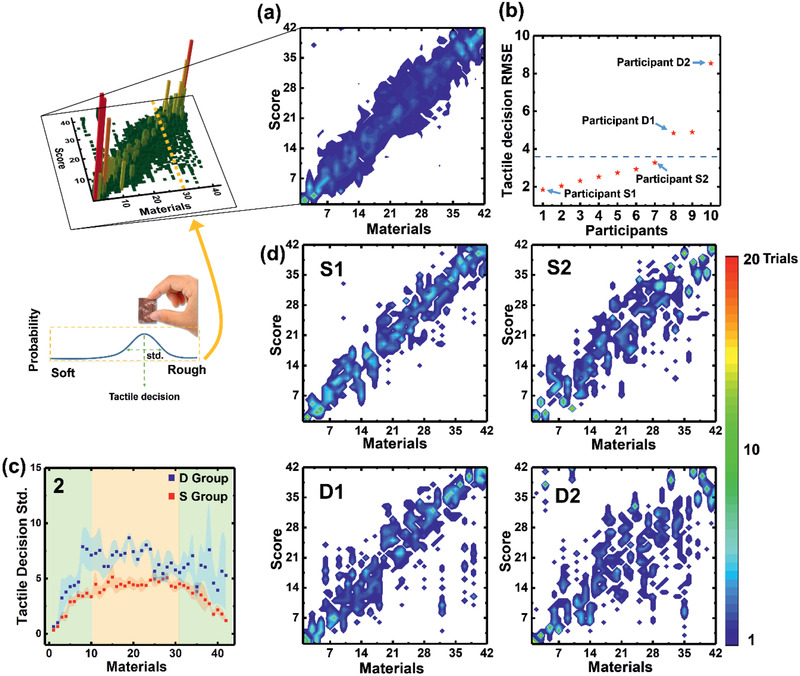
Human tactile decisions for 42 tactile samples. a) Averaged histogram of the tactile decisions of 10 participants; (a)‐1 and (a)‐2 are the graphical representations in 2D and 3D, respectively. b) Tactile decision difference (mean RMSE), that is, difference between the average decisions of each subject. Dashed horizontal line, which indicates the average of tactile decision RMSE divides the S (lower) and D (upper) groups. c) Tactile decision standard deviation values for the S (red, n = 7) and D (blue, n = 3) groups. Dots and shadows correspond to the group mean and variance, respectively. d) Example tactile decisions of the four participants identified in (b).

In this study, to design a human‐like tactile system, 42 materials were ranked from smoothest/softest to roughest by 10 participants. The samples were fabrics differing in surface morphology, thickness, and other characteristics (Figure S1e, Supporting Information). For the diversity of base materials, these were selected from a large library of general materials used in clothing, making them difficult to distinguish by humans. Especially, we considered cloths as tactile materials that represent softness and roughness the best among categories of LMT Haptic Texture Database.^[^ ^54^ ^]^ The participants were free to touch and slide their fingers (of both hands) over the tactile materials. They ranked them from 1 to 42, with 1 being the smoothest/softest and 42 being the roughest. The test results of all the participants were averaged, and the tactile materials were ordered accordingly (reference order). **Figure** **2**a shows a histogram for each tactile sample, where the color corresponds to the number of hits on the score. Figure 2b shows the tactile decision root‐mean‐square error (RMSE) between the reference values, which is calculated based on the mean decision of all participants for each material, and that of each participant (for more details, see Experimental Section). The participants ranked the samples differently, reflecting differences in tactile decision‐making. Seven participants made similar tactile decisions, that is, had tactile decision RMSE values lower than the averaged tactile decision RMSE (horizontal dashed line in Figure 2b); the remaining three participants had RMSE values that were higher than the averaged tactile decision RMSE. This indicates that human judgments are not consistent for ranking samples according to their tactile qualities. The participants in groups S and D made similar and dissimilar tactile decisions respectively, according to their tactile decision RMSE values relative to that of the average tactile decision (Figure 2b); participants with tactile decision RMSE values lower than the average tactile decision belonged to group S, whereas those with tactile decision RMSE values higher than that of the average tactile decision belonged to group D.

In addition to the tactile decision between humans, tactile confusion, which indicates the sensitivity of a human in sorting materials from No. 1 to No. 42 samples, is a critical individual feature of tactile decision. Figure 2c illustrates that the participants exhibited more tactile decision variance in the middle materials (in terms of rank; i.e., samples 11–30, shaded orange in Figure 2c) compared with side tactile materials (samples 1–10 and 31–42; shaded green in Figure 2c). Participants in group S had a smaller variance in trials, representing less tactile decision standard than those in group D. This implies that humans have different sensitivities for tactile materials. Because the tactile materials are not uniformly selected by considering the quantitative difference in the physical structure between materials, the middle materials may have a smaller physical difference than the side materials. This could result in tactile confusion difference with respect to materials and individuals.

To compare individual differences in tactile decisions in detail, a colored decision matrix was created, including the participants with the lowest (participant S1), largest (participant D2), and intermediate tactile decision RMSE values (participants S2 and D1) in Figure 2d. The averaged decision matrix (Figure 2a) was similar to that of participant S1, who had the smallest variance in tactile decisions (Figure 2d). Overall, the clearest decisions (smallest variances) were seen for samples with low scores (soft tactile sensation) or high scores (rough tactile sensation). The middle materials showed a wider distribution in scores, indicating greater decision confusion. The histograms of participants S2, D1, and D2 indicated clear decisions for the low‐ and high‐scoring materials, and decision confusion for the middle samples. Note that the scoring patterns for S2, D1, and D2 do not match the reference (averaged decision matrix in Figure 2a); each participant had their own unique response pattern. Therefore, to mimic human tactile cognition, a unique tactile system should be supported individually.

### 2.3 Artificial Sensor Systems for the Deep Learning Process

**Figure 3:**
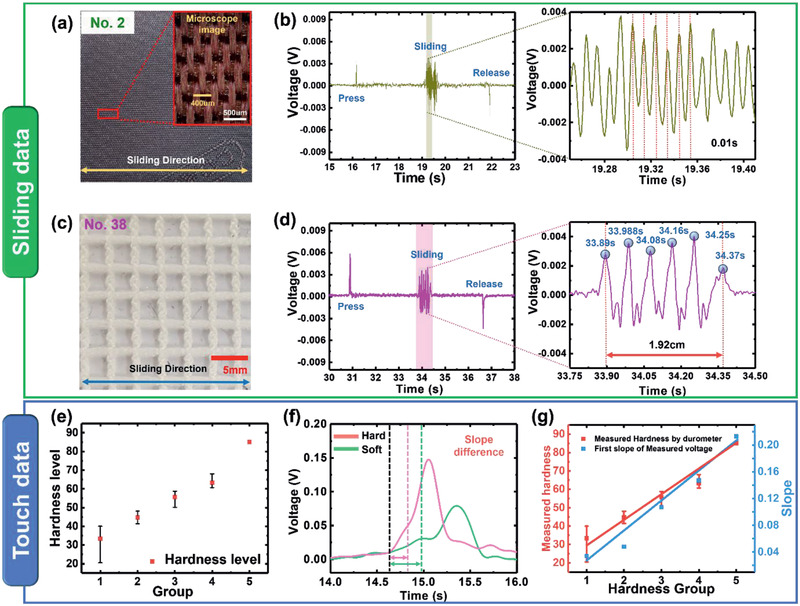
Information obtained from rubbing and touching the materials. a,c) Surface images of sample No. 2 and No. 38, respectively. b,d) Measured piezoelectric voltage by rubbing sample No. 2 and No. 38, respectively. e) Hardness level of 42 samples measured by commercialized durometer. f) Piezoelectric slope plotted using information obtained by touching hard or soft materials. g) Comparison of the first slope of piezoelectric voltage and measured hardness by durometer with increasing material hardness.

The multiarray tactile sensors to measure the surface information were made using piezoelectric materials. The details of the fabrication process were mentioned in the Experimental Section and the basic characteristics of our tactile sensor are shown in Figure S2, Supporting Information. A machine‐learning algorithm was applied to produce artificial tactile feeling among 42 test tactile materials. In this study, the touching and sliding signals were used in the analysis of surface information. The relationship between the characteristics of the material and the measured piezoelectric signal during sliding across a tactile material was investigated. The pattern width was obtained from the sliding signal. **Figure** **3**a,c shows the surface images of two different fabrics (No. 2 and No. 38). The fabric in (a) has a specific pattern, shown clearly in the inset. The pitch pattern of (a) along the sliding direction is about 400 µm. When the tactile sensing system slid across the surface of (a), complex piezoelectric signals were obtained (Figure 3b). The time interval between the measured piezoelectric signals was approximately 0.01 s (Figure 3b—right). The surface pattern pitch can be calculated by multiplying the velocity by the time interval. Because a sliding velocity of 4 cm s^−1^ was calculated by signals and the design factors of the sensor, the pitch of sample No. 2 was about 400 µm (4 cm s^−1^ × 0.01 s). The surface of sample No. 38 has a check pattern: the width of 5 pitches was 2 cm and the calculated value (1.92 cm) was almost identical to the actual value. The surface was easily deformed by shear and normal forces due to the softness of the fabric which caused the small difference (0.08 cm).

The hardness of a material, which is one of the major physical parameters informing the deep learning process in this study, can be easily measured for the touch data. The hardness of a fabric material was measured using a commercial durometer. The values for the 42 materials were categorized into five: 1: 20–40, 2: 41–50, 3: 51–60, 4: 61–70, and 5: 71–90 (Figure 3e). The hardness of fabric was determined based on the piezoelectric signals. The slope of the generated piezoelectric signal differed with the hardness level; the slope of the signal in the touched state is shown in Figure 3f. The durometer values of samples No. 2 and No. 38 were 85.1 and 24.9, respectively. Under the same test conditions, the harder the material, the steeper was the initial slope of the generated piezoelectric signal. The slope of the measured signal in Figure 3f shows that the hardness of fabric No. 2 was larger than that of sample No. 38, consistent with their durometer values. The measured piezoelectric slopes and durometer hardness are compared in Figure 3g. The analysis revealed that the initial slope was proportional to the durometer hardness of the fabric. Therefore, a relative hardness value can be obtained by the piezoelectric signals during the touching motion of the tactile sensor.

### 2.4 Deep Learning‐Based Tactile Machine for Tactile Discrimination

Based on the piezoelectric signals measured by touching and sliding the materials, the tactile decision system was designed by using a combination of neural network layers as shown in Figure 1. In this system, each parallel network was designed for extracting specific features and complex classification processing. The overall network was designed to mimic the cognitive processes engaged in by humans for tactile classification based on numerous types of tactile receptors.^[^ ^50^ ^]^ The end layer of the network is important when a machine is being trained with data, because it greatly affects the weight adjustment between networks. The fully connected layers were designed to be shrunk, to abstract the information and to allow the sample rank order based on human decisions to be reported.

**Figure 4:**
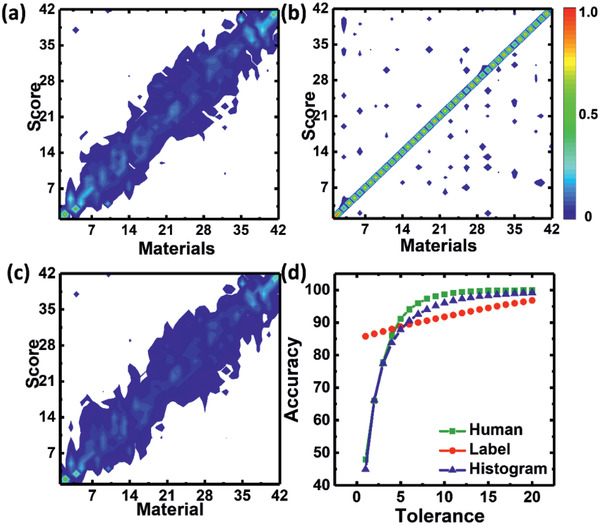
Deep learning for tactile decision‐making. a) Histogram of human tactile cognition. b) Tactile histogram of the machine trained using labels. c) Output layer of the proposed histogram‐based network for the 42 tactile materials with textures ranging from smooth/soft (bottom) to rough (top). d) Classification accuracy according to the tolerance bounds.

The typical method for training a neural network involves using labels that correspond to only one activated node among many nodes representing the trained tactile samples in the output layer. This approach has been widely adopted by artificial sensors to classify tactile materials. During the training process, our network was optimized to pick the correct tactile sample from among all the trained samples. To test the supervised learning based on labels, we trained the machine using the averaged tactile decisions as labels. The classification results are shown in the histogram in **Figure** **4**b by colored spots, which fall mostly along the diagonal. This indicates that the machine was trained successfully; it tended to pick the correct samples, that is, those along the diagonal. The machine did not exhibit decision confusion, which was observed in the human participants. Whereas the machine trained by labels made focused decisions on trained labels, the human participants did not make absolute (i.e., consistent) tactile decisions over multiple trials (Figure 4b), showing tactile confusion over a wide area. The decision pattern difference between humans and the machine is that the machine trained by labels makes incorrect judgments having scattered decisions far from the trained label, whereas humans make incorrect judgments on nearby materials.

### 2.5 Probabilistic Tactile Decisions Made by the Machine Trained Using the Human Tactile Decision Histogram

In this study, we propose a new approach to train a machine to make tactile decisions, based on a human tactile decision histogram. The nodes in the output layer represent the rank order of the tactile samples. To train the machine, we mapped the human tactile decision histogram to the output nodes. The histogram contains information regarding the averaged tactile decisions of the humans and the decision confusion, that is, variance in tactile decisions. Because the output nodes were sorted from smooth/soft to rough and were mapped using a histogram that had non‐zero values for multiple nodes, training on the relationships among tactile materials was possible.

We observed that the pattern of activation in the output nodes (Figure 4c) corresponded to the human tactile histogram (Figure 4a). The activation of the output nodes represents the tactile decisions (Figure 4b). Note that the output nodes were sorted in order from smooth/soft to rough, so that the activation pattern of the nodes represents the probability distribution of the tactile decisions. These probabilistic tactile decisions can be considered as trained tactile judgments made by the machine, because it was trained using the human tactile cognition histogram.

However, training the machine using only the histogram did not allow it to make definitive tactile decisions, because the output layer representing the tactile decisions included only the probability data for the 42 nodes. To enable a tactile decision to be made, an additional decision‐making ability was required prior to the output layer (Figure 1), based on Equation (1):

(1) $$\begin{array}{c} \mathrm{Expected}\;\mathrm{decision}=\mathrm{Round}\;\left(\mathrm{tactile}\;\mathrm{score}\times \mathrm{node}\;\mathrm{activation}\;\mathrm{level}\right) \end{array}$$

Additionally, to calculate the classification performance with consideration of decision confusion, the classification bound was adjusted by the tolerance level. Based on the mean score for each tactile sample according to the human histograms, the tolerance level was set as small or large. For example, a tolerance level of 2 allowed a distance of less than or equal to two ranks between the expected decision and the mean of human tactile decision, otherwise known as the classification error (Figure 1).

High classification accuracy is important for a machine to closely mimic human tactile cognition. Using supervised learning and training the machine with the human tactile histograms, it was possible to achieve human‐like tactile decisions. Based on the classification performance according to the tolerance level (Figure 4d), the machine trained on the human tactile histograms behaved similarly to humans. For the conventional classification which have a softmax with categorically labeled output makes the decision based on maximum likelihood; it takes the node having the highest value (maximum likelihood) in the output nodes as the decision.^[^ ^55^ ^]^ It is specialized for a classification to select one among the multiple candidates. However, since the target value (human decision histogram) in this study is not a unique value but a random variable which can be explained by probability distribution following Gaussian shape (see Figure 2a) and the output of the network is treated as the probability distribution, expected decision (Equation (1)) was applied to have one unique decision in both human decision and the proposed avatar system for the classification performance evaluation, especially for the accuracy.

Deep learning is used for various classification problems, including tactile classification.^[^ ^38^, ^39^, ^40^ ^]^ Various neural network structures have been proposed based on supervised learning with labels. The objective of those studies was to achieve higher classification accuracy. In this study, we did not focus on higher classification accuracy on a specific label, but on mimicking human tactile decision‐making. The tactile cognition test results of the individual participants (Figure 2d) showed that, distinct from tactile decision‐making per se, the tactile decisions of a given individual were reflected in both the overall tactile sample rankings and the degree of decision confusion (average tactile decision and variance of tactile decision, respectively).

### 2.6 Ability of the Machine to Mimic the Tactile Decisions of a Human Participant

**Figure 5:**
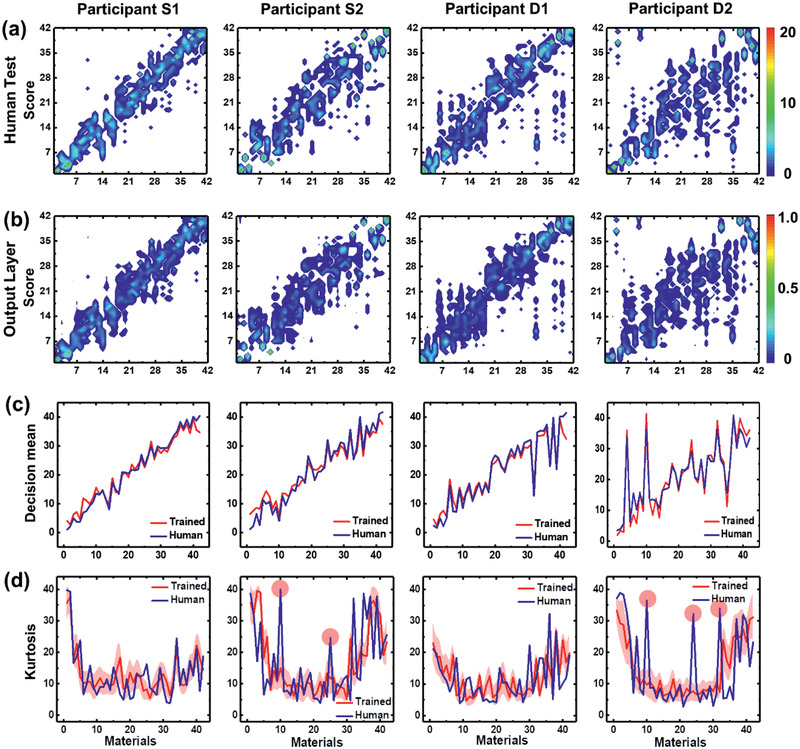
Fully trained network developed to mimic the tactile decisions made by humans. a) human and b) artificial tactile decisions for the four participants represented in Figure 4a. Analysis of the tactile decision similarity between the machine and human participants, based on c) mean and d) kurtosis values. Data for trained are presented as the mean ± Standard deviation.

We assessed whether the machine trained using human tactile histograms could represent the overall variance of tactile decision components for a given individual to make his/her tactile avatar. We trained the machine using a tactile histogram for 10 participants. The machine was trained according to the tactile decisions of each human participant (**Figure** **5**a and Figure S4a, Supporting Information), such that the decisions of the tactile avatar resembled those of the corresponding participant. The decision probability density for participants S1 to D2 was represented in the output layer of the neural network. Note that the individual histograms for participants S1 to D2 were preserved in the output layers (Figure 5b and Figure S4b, Supporting Information). The tactile decision‐making exclusive to individual participants was preserved well (Figure 5c) and the machine could mimic decision confusion, as reflected by the similar kurtosis values (Figure 5d). Although the decision probability density of the tactile avatar (i.e., machine) indicated less clarity in decision‐making (represented by blurred areas in the figure) compared with the corresponding human participant, it nevertheless resembled the human tactile histogram. This indicates that the machine was able to learn the tactile decisions of human participants. The results suggest that the tactile avatars behaved similarly to the corresponding human participants. Thus, the network was successful in mimicking human tactile sensing and processing, as reflected by the tactile histogram in the output layer.

### 2.7 Recognition of Untrained Tactile Materials by the Tactile Avatar

All materials have tactile properties, and new tactile materials continue to emerge; humans categorize new tactile materials based on prior experience. Using the typical supervised learning approach with labels, new tactile samples first need to be trained before they can be classified. However, it is inefficient and inconvenient to train every new tactile sample. The tactile avatar in this study was trained using human tactile decision histograms, considering both sensing and processing. In this manner, the avatar was able to make tactile decisions regarding untrained tactile samples. The tactile prediction performance for untrained tactile materials is illustrated in Figure S5b, Supporting Information, and discussed below.

**Figure 6:**
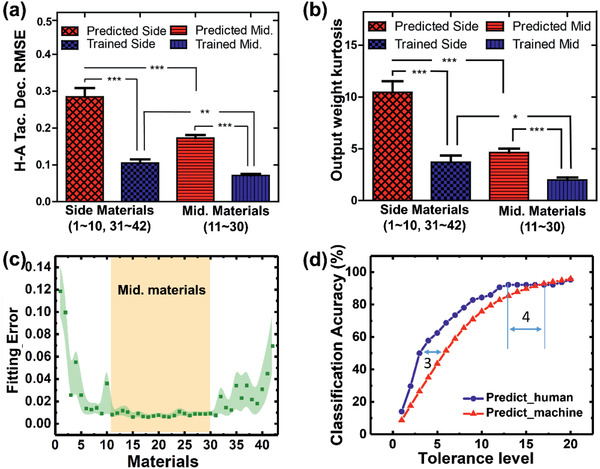
Untrained predictions designed to mimic human tactile decisions. a) Tactile decision difference between human and his/her paired avatar (H‐A tactile decision RMSE) for side and middle materials. b) Evaluation of decision confusion for trained (blue) and untrained (predicted) (red) cases, for side materials (materials 1–10 and 31–40) and middle materials (11–30). Data are presented as the mean ± SEM (side material; n = 22, mid. material; n = 20). T‐test were performed, and statistical significances are shown with an asterisk (*p < 0.05, **p < 0.005, ***p < 0.0001). c) Fitting error of the human tactile cognition data with the machine predictions for the middle materials. Dots and shadows correspond to the group mean and variance (n = 10), respectively. d) Classification accuracy by tolerance level.

We first measured the decision probability density of untrained tactile samples based on the level of activation of the output nodes. The decision error was given by the mean tactile decision difference (mean RMSE) for the trained and untrained (predicted) cases. Furthermore, kurtosis was calculated as a proxy for decision confusion. The tactile decisions of the machine were less similar to those of the humans in the untrained versus trained case, shown by the larger mean RMSE values for the human participants (**Figure** **6**a). Interestingly, the mean RMSEs of the middle samples had smaller errors than those of the materials with low or high ranks. Kurtosis was relatively low as well, for the middle materials (Figure 6b). This implies that the prediction of middle materials was more dependent on the trained tactile data. The avatar could make tactile decisions based on the signals arising from touching and sliding motions, and compare the signals to those of the trained tactile materials. Middle samples were more similar than low‐ and high‐scoring samples (evidenced by overlapping histograms). Thus, a newly experienced material could be compared to multiple trained materials.

A new tactile material can be classified based on the knowledge gained from previously trained materials. Considering individual tactile histograms to represent tactile knowledge, linearly combined histograms of previously trained tactile samples could provide a basis for processing new tactile experiences. Using this approach, we determined the degree of independence of each tactile material. The linear combination of pretrained tactile histograms that best fit the untrained tactile histogram was determined, and the corresponding RMSE was calculated. The difference in fit for both the low‐ and high‐scoring samples exceeded that of the middle samples (Figure 6c). This is because the middle samples were all relatively similar, whereas the high‐ and low‐scoring samples were more difficult for the machine to understand because of their relative uniqueness. This indicates that the tactile samples for which a clear ranking decision was reached by a human participant were more difficult to predict. In the experiment, it was impossible to include all the tactile materials experienced by humans in the real world. The limited tactile experience gained by the avatar, especially for the low‐ and high‐ranked materials, could be enhanced by training it using more materials, as per the actual experience of humans.

It is better to analyze the classification performance of the machine based on the middle materials because the ranks of these materials were predicted according to the tactile knowledge of trained data with relatively higher number of tactile materials sharing the same rank score. We compared the prediction performances of the machine and the humans. For the human prediction test, the participants were asked to predict the material's rank score having 3 anchor materials (#1, #21, and #42) (for more details, see Experimental Section). Because the participants already experienced the tactile materials, 1 year forgetting period was applied after the material rank‐scoring test. Figure 6d plots the prediction performance of the humans and the machine, which rises and then saturates. Overall, the performance of the human exceeded that of the machine. The tolerance difference to achieve the same accuracy was less than 4 tolerance level error, which is 9.5% considering 42 materials. Finally, as shown in Figure S6 and Movie S1, Supporting Information, based on the abovementioned signal processing and analysis, a tactile avatar system mimicking the tactile sensation of a specific user was developed. In a real‐time purpose, since the feedforwarding in the system for a real‐time use takes 0.9 ms in a GPU (NVIDIA Geforce GTX 1070 with Intel i7‐6700K) and 1.1 ms in a CPU (Intel i7‐6700K) processor respectively after taking sensing signal, it is feasible that it can be performed as a real‐time system, having millisecond delay. Since neuromorphic system mimics neural network in a chip,^[^ ^56^, ^57^ ^]^ this tactile avatar system on a neuromorphic system can reduce the time delay of the computation and minimize the size of the machine to perform in the realization.

## 3 Conclusion

The importance of digital applications will continue to increase, and tactile systems representing the interaction of hands with objects will become important. In this study, we developed a tactile avatar system, including a multiarray tactile sensor fabricated from piezoelectric materials, and a deep learning process based on human tactile cognition. The multiarray sensor had the same resolution as the tactile receptors on human skin. Prior to a complex signal processing process, parameters by sliding the materials and the material hardness were obtained directly from the measured electrical signal. However, it is challenging to obtain all surface information via a simple analysis. Therefore, we adopted a systematic approach to mimic human tactile cognition and thus enabled the tactile avatar to behave similarly to a human. In our system, the principal tactile components experienced by humans, that is, hardness and roughness, were processed by separable network layers that were designed for different functions; the network was trained using human tactile decision histograms. Even in the difficult case of predicting the rank of a previously unexperienced tactile sample, the developed tactile avatar system performed similarly to a human with the knowledge gained from previous experience. Future research should improve the ability of the tactile avatar to process tactile information, which will in turn allow machines to replace humans in virtual (i.e., VR and AR) spaces, such as online shopping malls.

## 4 Experimental Section

##### Fabrication of Tactile Sensor Based on P(VDF‐TrFE)

The fabrication process of the P(VDF‐TrFE)‐based tactile sensor was as follows. Polyimide film was used as the substrate of the sensor. A 5 × 6 array was patterned on a substrate by photolithography; the cell size was 1 × 1 mm^2^. The data from 30 sensing cells was enough for superior deep learning results. A 100‐nm‐thick Au layer and a 10‐nm‐thick Cr layer were deposited using a radio frequency magnetron sputtering system. Next, a 5 × 6 bottom electrode array of Au was obtained through a lift‐off process. The P(VDF‐TrFE) film was synthesized by a simple fabrication process as follows. Powdered P(VDF‐TrFE) (75/25) was dissolved in 2‐butanone to yield a 15 wt% P(VDF‐TrFE) solution. The mixture was stirred at 600 rpm without heating for 1 h to dissolve the P(VDF‐TrFE) powder. Once the mixture was dissolved completely, it was spin‐coated on the fabricated sensor array to function as the bottom electrode. Thermal annealing of P(VDF‐TrFE) at 130 °C for 2 h enabled beta‐phase crystallization, thereby inducing piezoelectricity. The annealed P(VDF‐TrFE) was finally cooled to room temperature outside the oven. The basic performance of the fabricated sensor is demonstrated in Figure S2, Supporting Information.

The interference of triboelectric effect in the fabrication tactile sensor was also considered. Generally, the triboelectric nanogenerator (TENG) device using the normal force exploited the structure maintaining the narrow gap of the tactile sensor like the spacer, arc‐shaped, or spring‐assisted separation structure.^[^ ^58^, ^59^, ^60^ ^]^ If there were no gap, a very poor electrical output performance was seen in TENG.^[^ ^61^ ^]^ Therefore, in the tactile sensor, there was no special structure making the gap for triboelectric effect and tried to minimize the gap by sticking the top electrode on a bottom substrate with UV glue.

##### Fabrication of the Dome Structure

Previously, special structures have been applied to the sensor for transferring the shear force to the normal force.^[^ ^62^, ^63^, ^64^, ^65^ ^]^ In this study, because the flat sensor could not interact with the surface structure, a special dome cover was also used to detect the surface information obtained by the shear force measurement. To fabricate this structure, a mold was 3D‐printed (ProJet 3500; 3D Systems, Rock Hill, SC, USA) for the dome, with the radius and height of 1 and 0.5 mm, respectively. The PDMS solution was produced by shaking the base oil and hardener (10:1). Air bubbles produced by shaking were removed over 30 min in a vacuum desiccator. The solution was poured into the 3D mold and left to harden for 12 h at 60 °C. The result of enhanced shear force sensitivity was demonstrated in Figure S3, Supporting Information.

##### Measurement Setup for Base Piezoelectric Signal

The basic sensing performance of the artificial tactile sensor was assessed using a digital oscilloscope and a low‐noise current preamplifier (Model SR570; Stanford Research Systems, Sunnyvale, CA, USA). The artificial tactile system measuring the piezoelectric signal during rubbing consisted of a multi‐source data acquisition system (PXIe‐5105; National Instruments, Austin, TX, USA) because multi‐channel sensing was required by the algorithm. The sampling rate during the measurements was 1000 s^−1^. The calibration was done to check some parameters such as deviation among cells or sensors before getting experimental data for the learning process.

##### Setup for Signal Processing and Tactile Sensing

The artificial sensing finger with touch and slide sensors was designed to resemble human tactile sensing. The tactile sensing system consisted of a tactile sensor and a 3D‐printed artificial finger with movement capabilities. The tactile sensor, made from piezoelectric material, was combined with the artificial finger (Figure S1d, Supporting Information) and equipment to enable movement (Figure S1a,b, Supporting Information). The sensing process comprised the touching, sliding, and release stages. Forty‐two fabric samples were used in this study (Figure S1e, Supporting Information), and the order of presentation thereof depended on the degree of feeling (smooth/soft to rough) reported by each participant. To collect surface information for the fabric samples, the system touched and slid the sample materials. After measuring the signal arising from the sensing system, the data were transferred to the signal processing system. At this stage, the tactile feeling level was determined, that is, smoother or less smooth relative to the judgment of the human participant.

##### Human Tactile Cognition Test

Ten participants (three females and seven males) of age 22–33 years participated in the experiments. The participants were asked to rank 42 tactile samples (Figure S1e, Supporting Information) from smooth/soft to rough; no time limit was imposed. The participants used both hands (all fingers) during the task; they were allowed to rub and touch the tactile materials with their fingertips, but not scratch them with their nails. Each participant completed 20 trials. The sample materials were randomly distributed at the beginning of each trial to prevent memorization. There was an interval of at least 4 h between repeated trials.

After more than 1 year, a tactile cognition test was performed with a limited number of samples. Three materials were presented as anchor materials with rank scores (#1, #21, and #42) for the participants. Based on the known scores of the anchors, the participants then performed the scoring of the eight given test materials. The anchor materials and the eight given test materials were chosen differently from the previous tactile cognition test.

##### Deep Learning Process

The tactile decision‐making process engaged in by the machine began with capturing tactile signals in two ways, that is, touching and sliding; these signals were initially processed through different neural network layers. A 1D convolution layer was used to extract the features of the touching data. A pooling layer down‐sampled and flattened the data for processing in conjunction with the sliding data. The sliding data were first transformed into the frequency domain, and then processed via a fully connected layer to achieve a complex, high‐dimensional classification (where the frequency features of tactile samples were distributed in the frequency domain). After processing the touching and sliding data in the neural network separately, they were combined to yield the overall tactile decision. In order to train human tactile decision in the probability distribution form (e.g., [0, 0.3, 0.4, 0.3, 0]), the output layer of the network following softmax activation was designed, which can express the output of the layer in form of a probability distribution; the summation of the total output is equal to 1 and each value in the node represents the likelihood. In addition, the mean‐square error loss function was utilized to fit the output of the network to the probability distribution of the human tactile decision.

For the performance comparison with the network trained by categorical labels, the categorically trained network was designed with the same network to the proposed network except for the loss function which is cross‐entropy loss function. The hyperparameters of the artificial network are described in **Table** **1**. Four TITAN Xp graphic cards (NVIDIA, Santa Clara, CA, USA) were employed using Compute Unified Device Architecture (CUDA) and the Python (version 3.7; https://www.python.org) package TensorFlow (Google Brain, Mountain View, CA, USA).

**Table 1:** Hyperparameters of the artificial neural network

Hyperparameter	Type	Value	Type	Value
Input layer	Touch signal (time [ms])	400	Sliding signal (1–500 Hz)	500
Parallel hidden layer	1D convolution	Layers: 3, kernels: 4 ReLU	Fully connected	400, ReLU
	Max pooling	4	Fully connected	300, ReLU
Combined hidden layer	Fully connected		400, ReLU	
	Fully connected		200, ReLU	
Output layer	Fully connected		42, Softmax	
Optimizer	Stochastic gradient descent		Decay: 10^−6^ Momentum: 0.9	
Loss function	Mean‐square error for proposed Avatar system Cross‐entropy for categorically labeled system			

##### Tactile Decision Difference and Tactile Confusion Calculation

The difference of a representative tactile decision was calculated by averaging decision between all participants and individual participants as follows:

(2) $$\begin{array}{cc} & \mathrm{Tactile}\;\mathrm{Decision}\;\mathrm{RMSE}\left(k\right)\;\\ & \;=\sqrt{\frac{1}{K}\sum_{k}^{K}{\left(\frac{1}{S}\frac{1}{I}\sum_{s}^{S}\sum_{i}^{I}Tac^{i}\left(s,m\right)-\frac{1}{I}\sum_{i}^{I}Tac^{i}\left(s,m\right)\right)}^{2}}\; \end{array}$$

where *Tac^i^*(*s*,*m*) is the tactile decision in *i*th trial (*i* = [1,2, …, *I*]) for participants *s* (*s* = [1,2, …, *S*]) and material *m* (*m* = [1,2, …, *M*]), and *k* is the variable index for analysis with respect to participants and material for analysis (i.e., *k* = *m* and *k* = *s*, respectively). To compare the tactile decision difference between a human and his/her paired avatar, the tactile decision was calculated as

(3) $$\begin{array}{cc} & H-A\;\mathrm{Tactile}\;\mathrm{Decision}\;\mathrm{RMSE}\left(m\right)\;\\ & \;=\sqrt{\frac{1}{S}\sum_{s}^{S}{\left(\frac{1}{I}\sum_{i}^{I}Tac^{i}\left(s,m\right)-\frac{1}{I}\sum_{i}^{I}{\bar{Tac}}^{i}\left(s,m\right)\right)}^{2}}\; \end{array}$$

where ${\bar{Tac}}^{i}\left(s,m\right)$ is tactile decision of an avatar. The tactile confusion for human participants was calculated as

(4) $$\mathrm{Tactile}\;\mathrm{decision}\;\mathrm{std}.\left(s,m\right)\;=\;\sqrt{\frac{1}{I}\sum_{i}^{I}{\left(Tac^{i}\left(s,m\right)\;-\;\frac{1}{I}\sum_{i}^{I}Tac^{i}\left(s,m\right)\right)}^{2}}\;$$

##### Statistical Analysis

Tactile decision standard deviation values for the S (red, n = 7) and D (blue, n = 3) groups were presented as the mean (±) and variance. Graphical presentation of the data was performed by OriginPro software (OriginLab Corporation, Northampton, USA). The data for the comparison between predicted and trained were presented as the mean (±) standard error of the mean (SEM) with the samples (side material; n = 22, middle material; n = 20). Two‐tailed t‐test was performed, and the criterion for statistical significance was set at * for *p* < 0.05, ** for *p* < 0.005, and *** for *p* < 0.0001. The analysis was conducted using Prism software (GraphPad Software, Inc., La Jolla, USA).

## Conflict of Interest

The authors declare no conflict of interest.

## Supporting information

Supporting Information

Supporting Movie 1
