# Supplementary material for: Tactile Avatar: Tactile Sensing System Mimicking Human Tactile Cognition
Source: Adv Sci (Weinh). 2021 Feb 8;8(7):2002362. doi: 10.1002/advs.202002362 (PMC8024994; doi:10.1002/advs.202002362)
Supplement: Supplementary file 1 — Supporting Information [file ADVS-8-2002362-s001.pdf]

© 2020 Wiley-VCH GmbH

ADVANCED SCIENCE
Open Access

Supporting Information

for *Adv. Sci.*, DOI: 10.1002/advs.202002362

Tactile avatar: Tactile sensing system mimicking human tactile cognition

Kyungsoo Kim, Minkyung Sim, Sung-Ho Lim, Dongsu Kim, Doyoung Lee, Kwon Sik Shin, Cheil Moon, Ji-Woong Choi, and Jae Eun Jang**

Supporting Information

Figure S1. The setup of tactile sensor array and moving equipment.

Figure S2. Basic performance of the piezoelectric tactile sensor.

Figure S3. Applied dome structure and enhanced sensitivity.

Figure S4. Deep learning process trained by human tactile cognition histogram and tactile classification with tolerance level.

Figure S5. Recording data separation for fully trained and tactile precision.

Figure S6. The tactile avatar system.

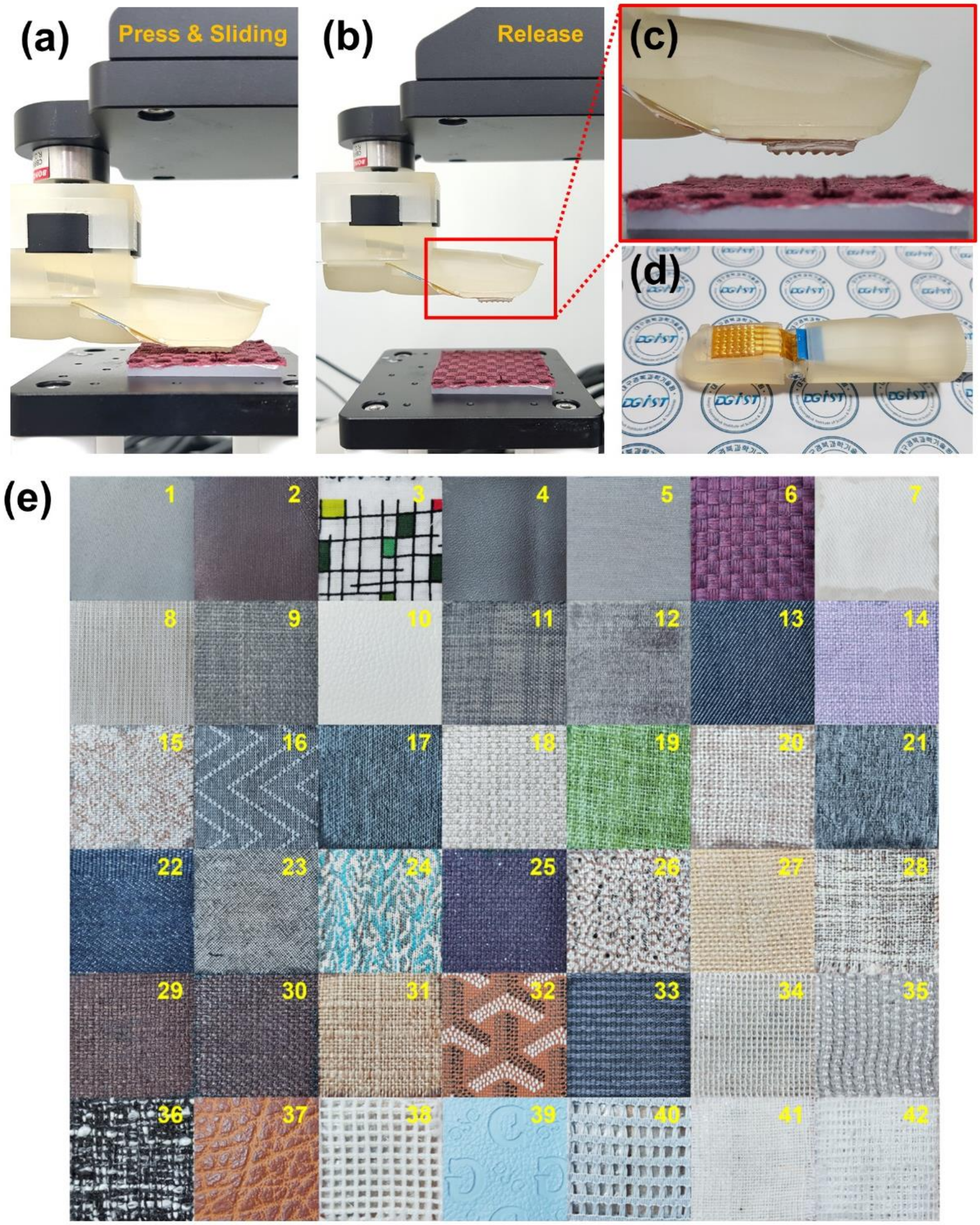

Figure S1. (a) the touching, sliding, (b) release state of customized moving equipment, (c) the magnitude image of tactile sensor and rubbed surface material, (d) the tactile sensor with 3D-printed artificial finger model, (e) 42 of fabric used for this study.

- Our tactile avatar system consisted of a piezoelectric tactile sensor with a three-dimensionally printed artificial finger, XYZ linear translation stage, and signal processing capability. A piezoelectric signal was obtained via touching and sliding motions. The touching signal was related to hardness, and the high-frequency sliding signal provided surface information on the samples. The information was transferred for processing and the roughness level of the samples was determined.
- In this study, 42 fabric samples were evaluated by human participants (Fig. S1(e)). The fabric samples are often found in daily life. The numbers of the samples were in accordance with the roughness level (softest sample = No. 1; roughest sample = No. 42).

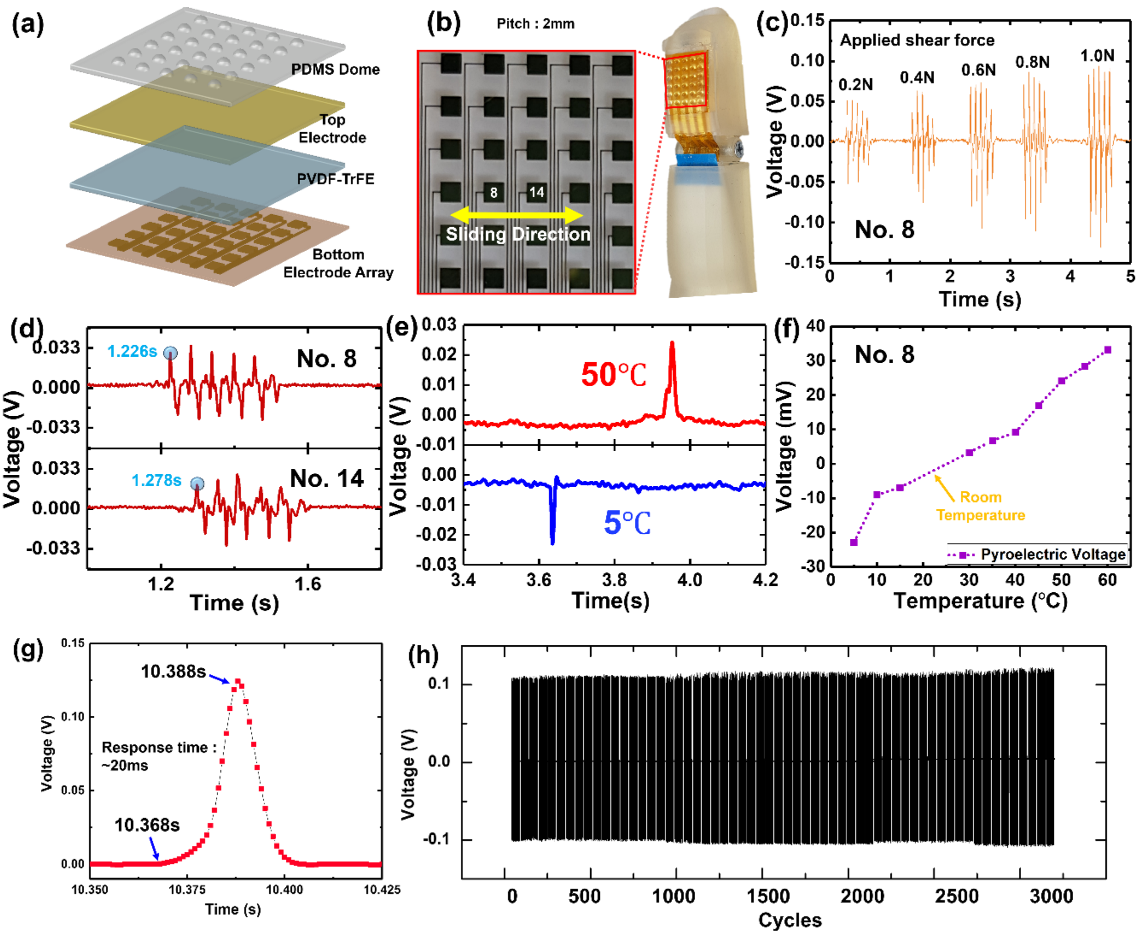

Figure S2. Basic performance of the piezoelectric tactile sensor. (a) Schematic and (b) 5 × 6 tactile sensing cell of the fabricated tactile sensor and the artificial tactile finger design with tactile sensor. (c) Piezoelectric voltage produced by rubbing, with increasing rubbing force. (d) Piezoelectric signals of two sequentially arrayed sensing cells to measure the rubbing velocity. (e) Pyroelectric voltage at low and high temperature, and (f) temperature sensitivity by the pyroelectric effect, (g) the response time and (h) the stability of tactile sensor

- Figure S2(a) schematically illustrates the tactile sensor. It consisted of a pair of electrodes, P(VDF-TrFE) as the primary material, and a dome structure to amplify the piezoelectric signal, similar to a finger print. The sensor had a 1 × 1 mm^2 cell size and 2-mm pitch (Fig. S2(b)). Because these dimensions are similar to the spatial resolution of human fingertips,[22,46] we think that current or a little higher resolution design is enough for tactile sensor. The total area of the tactile sensor was about 1 cm^2. The tactile sensor array was embedded in a 3D-printed fingertip model to assess the electromechanical characteristics and the feasibility of the sensor. The fingertip equipped with the tactile sensor was installed on an XYZ linear translation stage. Details of the sensing process are given in

Supporting information (Fig. S1). Figure S2(c)–(f) show the piezoelectric and pyroelectric behaviors of the P(VDF-TrFE) material used in the tactile sensor. The increasing of piezoelectric voltage (Fig. S2(c)) was measured by sliding stepwise across the surface of a given material with increasing applied force. Sliding velocity is another important surface parameter because velocity sensing is a problem with other sensors. Figure S2(d) shows the piezoelectric signals arising from two seriate sensor cells as the sensor slid across a fabric. The multiarray structure of the sensor could readily determine the sliding velocity based on the measured piezoelectric signal without any speedometer. Therefore, our sensor could obtain surface information regardless of the various sliding velocities. Because the sliding direction and pitch of the sensor cells were fixed, the velocity could be calculated from the piezoelectric signal of each cell. For example, in Fig. S2(d), the time interval between two peaks is 0.052 s and the moving distance is 2 mm. The calculated velocity is then 3.85 cm/s, which is in good agreement with the actual velocity of the XYZ stage.

- Figure S2(e) shows the pyroelectric behavior of P(VDF-TrFE) as a function of temperature. Increasing or decreasing the ambient temperature caused thermal expansion or compression, which changed the structure of the piezoelectric material. This change resulted in polarization of the material and generation of a pyroelectric voltage (Fig. S2(e)). The voltage increased from negative to positive with increasing temperature (Fig. S2(f)). Notably, the induced voltage was zero when a fabric sample maintains its temperature at the same level as the room temperature, i.e., it indicates that the sensor detected a temperature difference rather than an absolute temperature, similar to human skin. Because the main decision parameter is human tactile sensation, it is preferable to generate an artificial tactile feeling based on a deep learning process. The response time of tactile sensor is 20 ms in Fig. S2(g) and the performance of voltage generation was maintained during 3000 cycles when the force of 1.2N is applied in Fig. S2(h).

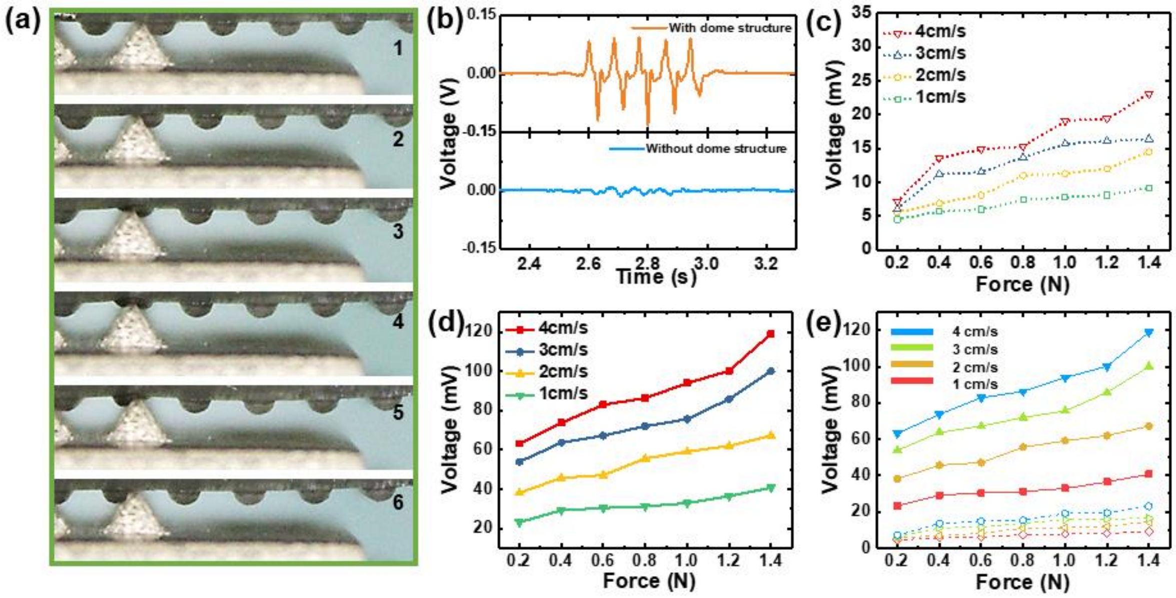

Figure S3. (a) 6 images of the process when the triangle materials designed by 3D printer were rubbed on the dome structure (b) measured piezoelectric voltage with/without dome structure, the shear force sensitivity (c) without (dashed lines) and (d) with (solid lines) the dome structure, and (e) a graph comparing the two cases.

- In this study, a polydimethylsiloxane (PDMS) dome structure was installed on top of the tactile sensor to amplify the piezoelectric signal and thereby obtain more precise information regarding the material surface. The six images in Fig. S3(a) show the tactile sensor sliding across a pyramidal object. As shown in these figures, the dome was deformed, and transferred the applied force well to the piezoelectric sensing cell when the surface structure of the tactile material contacted the dome structure. To confirm the effect of dome structure, the shear force voltage measurement was carried out. Figure S3(b) shows the different initial piezoelectric signals for the simple planar sensor structure and the sensor with the dome structure. The results from Fig. S3(c) to (e) indicated that the higher dome structure, the higher shear force sensitivity was observed.

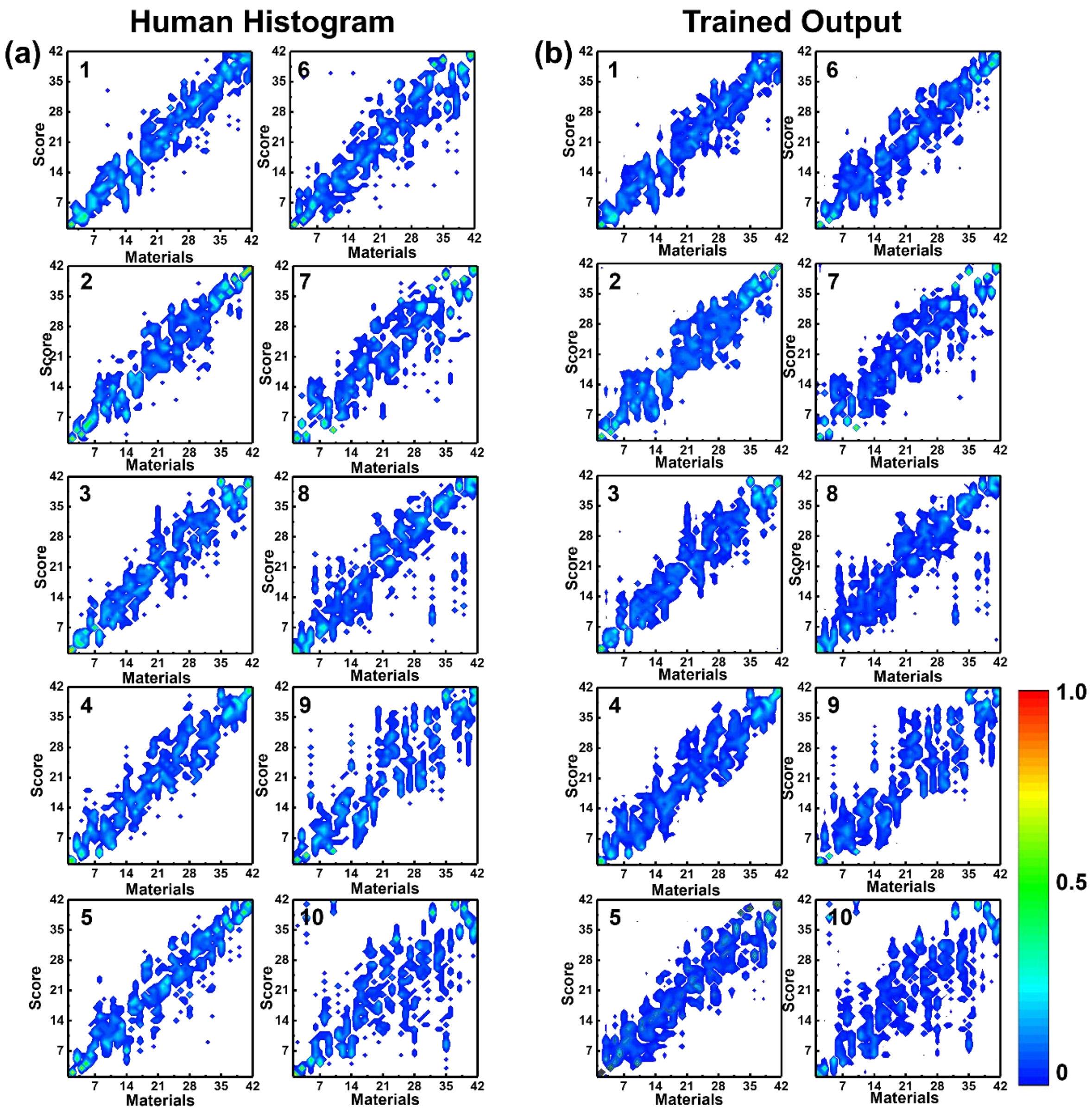

Figure S4. (a) Tactile histogram of a human participant and (b) the corresponding output data of the tactile avatar.

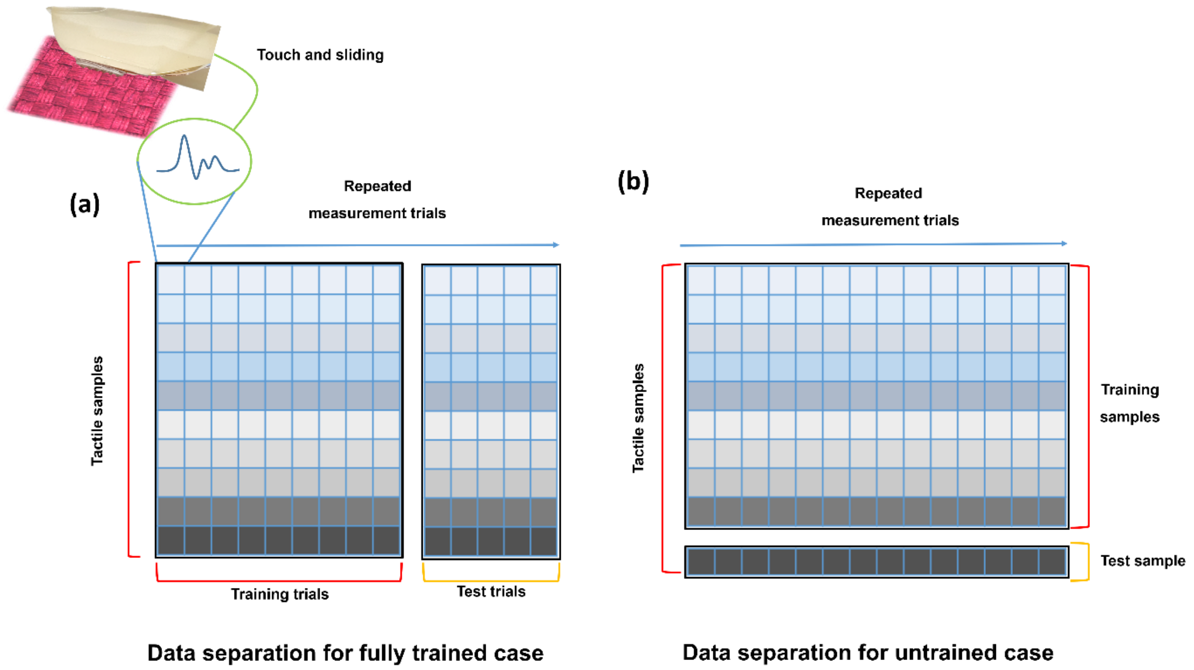

Figure S5. Data separation for (a) fully trained and (b) untrained cases.

To test the machine performance using a limited number of fully trained and untrained tactile samples, we separated the data in two ways. For the fully trained network, samples were separated into training and test sets over repeated trials, while for the untrained materials, one of the tactile samples was not included in the training set. The untrained tactile sample data were included in the test set to evaluate the prediction performance of the tactile avatar. In the experiment, we trained the avatar with 41 of the 42 samples and tested it with the untrained samples. For example, in one trial, tactile samples 1–41 were used for training and sample 42 was tested as the untrained sample. In this manner, all tactile materials were used as untrained samples.

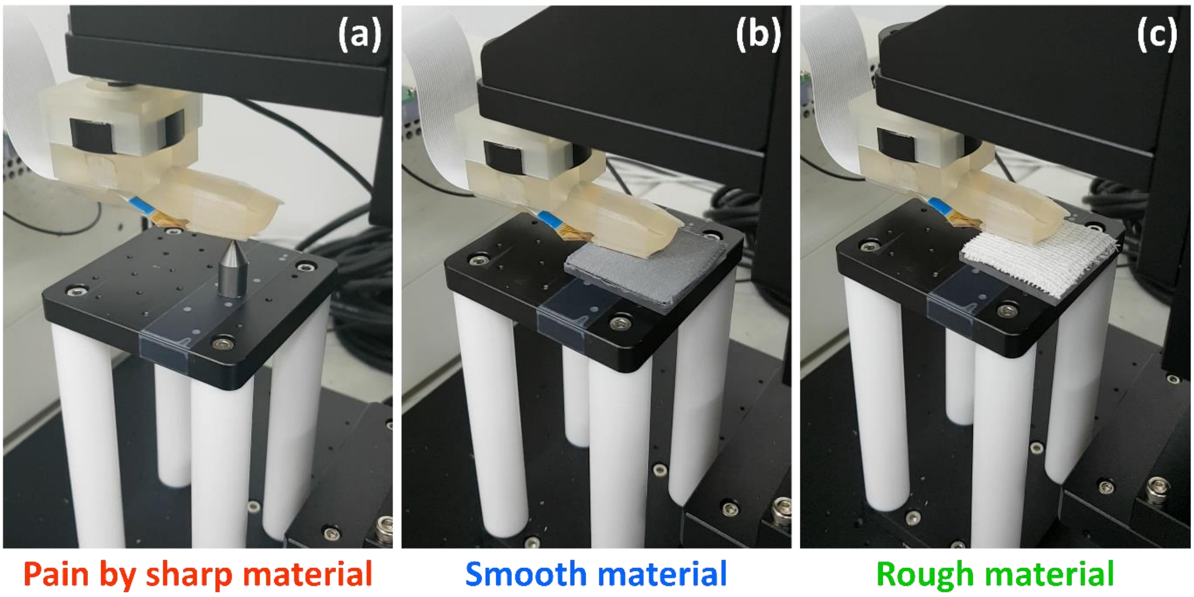

Figure S6. The tactile avatar system. (a) pain warning signal when the artificial finger with tactile sensor touched the sharp material was generated. (b) smooth/soft or (c) rough feeling signal was generated depending on tactile material feature when the material is not sharp after touching and sliding.

In this study, the tactile avatar system was designed to generate artificial tactile feeling in accordance with the feeling of a specific person (master). First, when the artificial finger touches the unknown material, this system determines whether the material is sharp or not. If sharp, the system generates a warning signal and lifts the finger back. If not sharp, the finger touches and slides on the material, and finally, produces the smooth/soft or rough tactile sensations. Since this sensation was trained by the master's tactile feeling, this system can be operated as a tactile avatar of the master. Like the master, the avatar system can generate feeling, 'smooth/soft' or 'rough' to unexperienced materials. A supporting movie M1 shows the operation of a tactile avatar system to a sharp, a smooth/soft or a rough material.
